# Glider observations of interleaving layers beneath the Kuroshio primary velocity core east of Taiwan and analyses of underlying dynamics

**DOI:** 10.1038/s41598-019-47912-z

**Published:** 2019-08-06

**Authors:** Sen Jan, Shih-Hong Wang, Kai-Chieh Yang, Yiing Jang Yang, Ming-Huei Chang

**Affiliations:** Institute of Oceanography, National Taiwan University, Taipei City, Taiwan

**Keywords:** Environmental impact, Physical oceanography

## Abstract

Submesoscale interleaving layers are caused by lateral intrusions of dissimilar water masses in frontal zones, which are significant processes in shaping physical, biogeochemical, and ecological parameters in the ocean. Possible interleaving layers were sometimes observed by ship-based conductivity-temperature-depth (CTD) surveys with coarse spacing between adjacent stations in the Kuroshio region east of Taiwan but have never been examined dynamically. Here we show the characteristics of interleaving layers observed by a Seaglider with two repeated hydrographic surveys along a triangle track east of Taiwan from December 2016 to March 2017. Salinity profiles indicate that prominent interleaving layers appeared in the intermediate layer (approximately 500–800 m) with vertical and horizontal length scales of O(50) m and O(10–100) km, respectively, during our observations. A dipole eddy pair and a relatively large anticyclonic eddy impinged on the Kuroshio during the first and second surveys, respectively, which brought certain impacts on the interleaving motion as the eddy potentially altered the density slope across the Kuroshio. The associated instability analysis and the Turner angle suggest that the double diffusive instability is the primary driving mechanism for the development of interleaving layers.

## Introduction

Submesoscale processes in the ocean have been widely studied for not only advancing our understanding of their dynamics in transferring energy from large- to small-scale oceanic motions but also for parameterizing their effects in large-scale ocean circulation models^1–3^. These processes also play certain physical roles in shaping marine chemistry and ecosystems^4,5^. Considering the significant influences of meso- to submesoscale processes on physical, biogeochemical, and ecological conditions in the ocean, comprehensive *in situ* observations have been conducted in the western boundary current of the North Pacific, i.e., the Kuroshio, since September 2012^6^, and the observational data have considerably improved our understanding of meso- and submesoscale processes in the Kuroshio east of Taiwan and vicinity^6–11^. The associated variability of the Kuroshio is mostly caused by the impingement of westward migrating mesoscale eddies of both signs and the topography that guides the eddy’s propagation and partially blocks the poleward movement of the eddy in a deep layer after the eddy encounters the offshore side of the Kuroshio.

**Figure 1:**
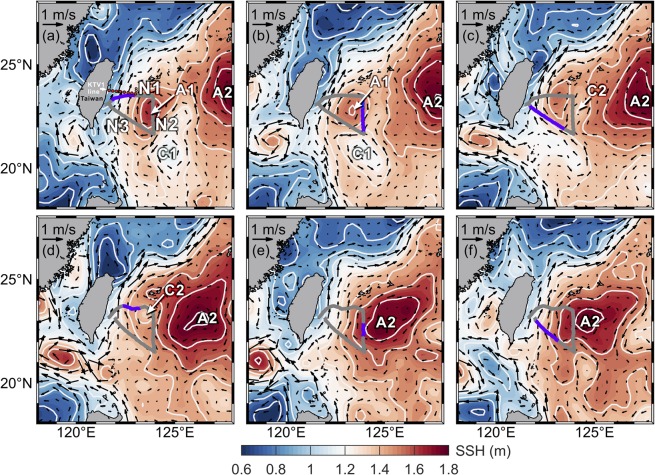
AVISO SSH (color shaded) and associated absolute geostrophic velocity (arrows) during the glider samplings. (**a**) 17–23 December 2016, (**b**) 27 December 2016–2 January 2017, (**c**) 6–12 January 2017, (**d**) 19–25 January 2017, (**e**) 8–14 February 2017, and (**f**) 24 February–2 March 2017. The contour interval for SSH is 0.2 m (white lines). The gray line indicates the glider’s track. The purple segment overlaid on the gray line indicates glider samplings during the period of composite SSH in each panel. East of Taiwan, cyclonic eddies are indicated by C1 and C2, and anticyclonic eddies are indicated by A1 and A2. The eight stations for repeated hydragraphic and velocity measurements at the KTV1 line are indicated by red dots in (**a**).

The topography east of Taiwan is an ~5000 m deep basin (Huatung Basin) connecting the broad western North Pacific (Fig. 1). To the north of the deep basin, the Ryukyu Islands Arc stretching from the southwest of Japan to the northeast of Taiwan behaves as a dynamic barrier, which prevents direct impingement of westward migrating eddies onto the continental shelf in the East China Sea but dictates the movement of eddies, particularly anticyclones, moving southwestward to the deep basin east of Taiwan^12^. Therefore, as the Kuroshio flows poleward along the east coast of Taiwan, the frequent impingement of mesoscale eddies can cause O(50–100) km zonal shifts in the Kuroshio’s maximum velocity axis with a synoptic time variation in its velocity structure and O(10) Sv (1 Sv = 10^6^ m^3^ s^−1^) variation in the mean volume transport (~20 Sv). This variability potentially converts energy from mesoscale to submesoscale processes, which creates density slope tilting and water mass exchange in the Kuroshio^7,8^ and a deep counter current beneath the Kuroshio^9^ through eddy-Kuroshio interactions. The physical mechanism underlying these meso- to submesoscale variations is crucial to the redistribution and dissipation of ocean energy in the western boundary region^2^.

Among the aforementioned submesoscale processes in the Kuroshio, interleaving layers between two different water masses (also called thermohaline intrusions), indicated by changes in the sign of vertical temperature or salinity gradients, particularly in the intermediate layer, have sometimes been found by shipbased conductivity-temperature-depth (CTD) observations at some hydrographic stations in the Kuroshio^6^. Indeed, interleaving layers have been commonly observed in many frontal zones in the world ocean (Table 1). For example, the front between Southern Ocean and Antarctic Circumpolar Current waters (i.e., Antarctic Polar Front) in the western Scotia Sea and the Drake Passage^13–15^, between cold and fresh waters from the subpolar region and North Pacific Central Water in the North Pacific subtropical frontal zone^1,16,17^, between brackish North Pacific and saline South Pacific waters in the western Equatorial Pacific^18^, between cold Arctic Ocean water and Atlantic water in the Arctic Ocean^19–21^, in the Agulhas Current^22^, and between Oyasho and Kuroshio Extension waters east of Japan^23^. Each interleaving layer in these frontal zones has its own characteristic, but the formation mechanism essentially starts from finite disturbances in the front of two dissimilar water masses, which trigger lateral intrusions from one to the other water (see Supplementary Fig. S1). The following growth of the interleaving is attributed to either the double diffusion^21^ or the advective driven intrusion through the cross-front velocity perturbations^22^. The associated driving mechanism of double diffusive intrusions is the salt fingering and diffusive convection generated subsequently with those initial disturbances. The alternative appearance of salt fingering and diffusive convection in the stratified layers generates convergence or divergence in the vertical buoyancy fluxes and, in turn, stretches the intrusions into distinct interleaving structures^1,14,15,21^ (Supplementary Fig. S1). In contrast, the advective intrusion is dominated by the cross-front inertial velocity anomalies through the baroclinic instability^22^.

**Table 1:** Summary of the characteristics of observed interleaving layers.

Region	Depth range (m)	Thickness (m)	Length (km)
Drake Passage – Arctic Polar Front^13–15^	100–300	50–100	1
Central Equatorial Pacific – Equatorial Salinity Front^34^	150–200	≦10	<100
North Pacific Subtropical Frontal Zone^1,16,17^	20–140	10	5
Western Equatorial Pacific^18^	80–240	20	100–200
Arctic Ocean^19–21^	Any	10–100	Entire basin
Agulhas Current^22^	500–1200	200	10
Kuroshio Extension Front^23^	150–500	10–100	N/A

The importance of examining the interleaving in the Kuroshio is that it provides a mechanism for the transformation of water mass properties, which can alter the density slope across the Kuroshio and, in turn, the associated velocity structure and throughflow volume transport. However, the spacing of adjacent CTD stations^6^ (~20 km) is too coarse to resolve the horizontal coherence of the interleaving layers and associated dynamics. The existence of this submesoscale structure indicates potential water mass exchange between South China Sea Water, Kuroshio Water or North Pacific Water in the Kuroshio. The characteristics of interleaving layers in the Kuroshio east of Taiwan and the formation of the interleaving have yet to be examined. To the best of our knowledge, this study serves as the first effort to quantify and examine the interleaving layers in the Kuroshio east of Taiwan using high-resolution hydrographic data collected by autonomous underwater vehicle (Kongsberg’s Seaglider in this study). The Seaglider was navigated along the three sections of a triangle track east of Taiwan (Methods). Two repeated surveys, the first and second surveys, were completed from 15 December 2016 to 14 January 2017 and from 14 January 2017 to 5 March 2017, respectively (Methods).

## Results

### Mesoscale features of sea surface height during the observations

The 7-day composite sea surface height (SSH) obtained from the Archiving, Validation, and Interpretation of Satellite Data in Oceanography (AVISO) provides synoptic oceanic features during our glider observations (Fig. 1). After the launching of the glider near the southernmost of Taiwan on 8 December 2016, a dipole eddy pair (A1 and C1 in Fig. 1) encountered the offshore side of the Kuroshio (Fig. 1a). The hydrographic samplings along the transect N1 captured the influence of the anticyclonic eddy (A1 in Fig. 1) of the eddy pair. The dipole eddy pair migrated ~50 km westward during the sampling along the transect N2, where it was mostly influenced by the eastern half of the anticyclonic eddy A1 as well as the westward-flowing confluent current of the eddy pair at ~22°N (Fig. 1b). The confluent flow continuously affected the velocity and hydrography at the transect N3 (Fig. 1c). During the second repeated survey at N1, a small, short-lived cyclonic eddy (C2 in Fig. 1) with diameter of only ~100 km appeared on the eastern half of N1 (Fig. 1d). The rest of the second survey was primarily influenced by a larger anticyclone with a diameter of ~400 km (A2 in Fig. 1e,f).

The westward-propagating mesoscale eddy is a plausible albeit debated mechanism transporting North Pacific water from its origin to the eastern flank of the Kuroshio. The inner core of an eddy, defined by the contour of zero relative vorticity, may comprise only water mass from its origin, and the outer ring of the eddy may contain a mixture of ambient water from throughout the eddy’s life^24^. Alternatively, the original water mass can be trapped and transported independently by propagating mesoscale eddies^25^. Regardless of this debate, the encountering of North Pacific water with Kuroshio water potentially creates fronts between the two water masses or alters the density slope across the Kuroshio, which provide basic conditions for the interleaving layers observed in this study.

### Water masses in the Kuroshio and carried by eddies

**Figure 2:**
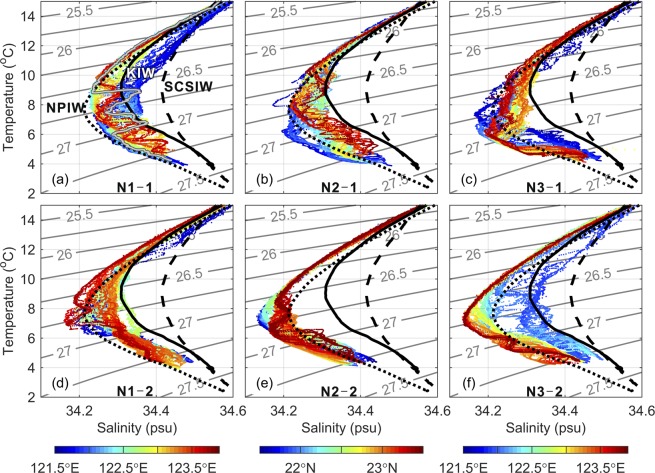
Temperature versus salinity (T-S) diagrams obtained from our glider observations. T-S diagrams of the first survey at (**a**) N1, (**b**) N2, and (**c**) N3 and the second survey at (**d**) N1, (**e**) N2, and (**f**) N3. Characteristic T-S curves of NPIW, KIW, and SCSIW are plotted by short-dashed, solid, and long-dashed lines, respectively, on each panel. The gray T-S curve (obtained at 122.25°E) showing a zigzag pattern in (**a**) is an example demonstrating interleaving layers between NPIW and KIW in the range σ_θ_ ~26.5–27.0 kg m^−3^.

The characteristics of water masses in the intermediate layer of the Kuroshio off Taiwan have been thoroughly investigated previously^7^. Using the glider observations, Fig. 2 illustrates the temperature versus salinity diagram (T-S diagram) for intermediate waters in each transect during each survey. The determination of the coordinates of the CTD samplings in the water is described in Methods. The intermediate water in the Kuroshio typically contains South China Sea Intermediate Water (SCSIW) near the east coast of Taiwan, North Pacific Intermediate Water (NPIW) appearing frequently on the offshore side of the Kuroshio, and Kuroshio Intermediate Water (KIW) carried by the Kuroshio itself from its origin^7^. The characteristic T-S curves of SCSIW, NPIW, and KIW^7^ are plotted on the T-S diagram in Fig. 2. These characteristic T-S curves were obtained from the average of historical CTD data at three selected regions^7^ shown in Supplementary Fig. S2. The three water masses are characterized by their ranges and means of salinity minima (${S}_{{\rm{\min }}}$, ${\bar{{\rm{S}}}}_{{\rm{\min }}}$), which are (34.17–34.26, 34.22) for NPIW, (34.26–34.36, 34.31) for KIW, and (34.30–34.43, 34.40) for SCSIW.

The T-S diagram suggests that intermediate waters primarily consisted of NPIW and KIW and their mixture in all transects during the first survey (Fig. 2a–c). The zigzag T-S curve, which is a necessary anatomy of interleaving layers, is seen in many of the T-S profiles on the three transects, e.g., the gray curve in Fig. 2a. SCSIW was rarely observed during the first survey; instead, a mixed water mass of KIW and SCSIW appeared near the onshore end of N1 and N3, between σ_θ_ = 26.0 and 26.8 kg m^−3^ (Fig. 2a,c).

**Figure 3:**
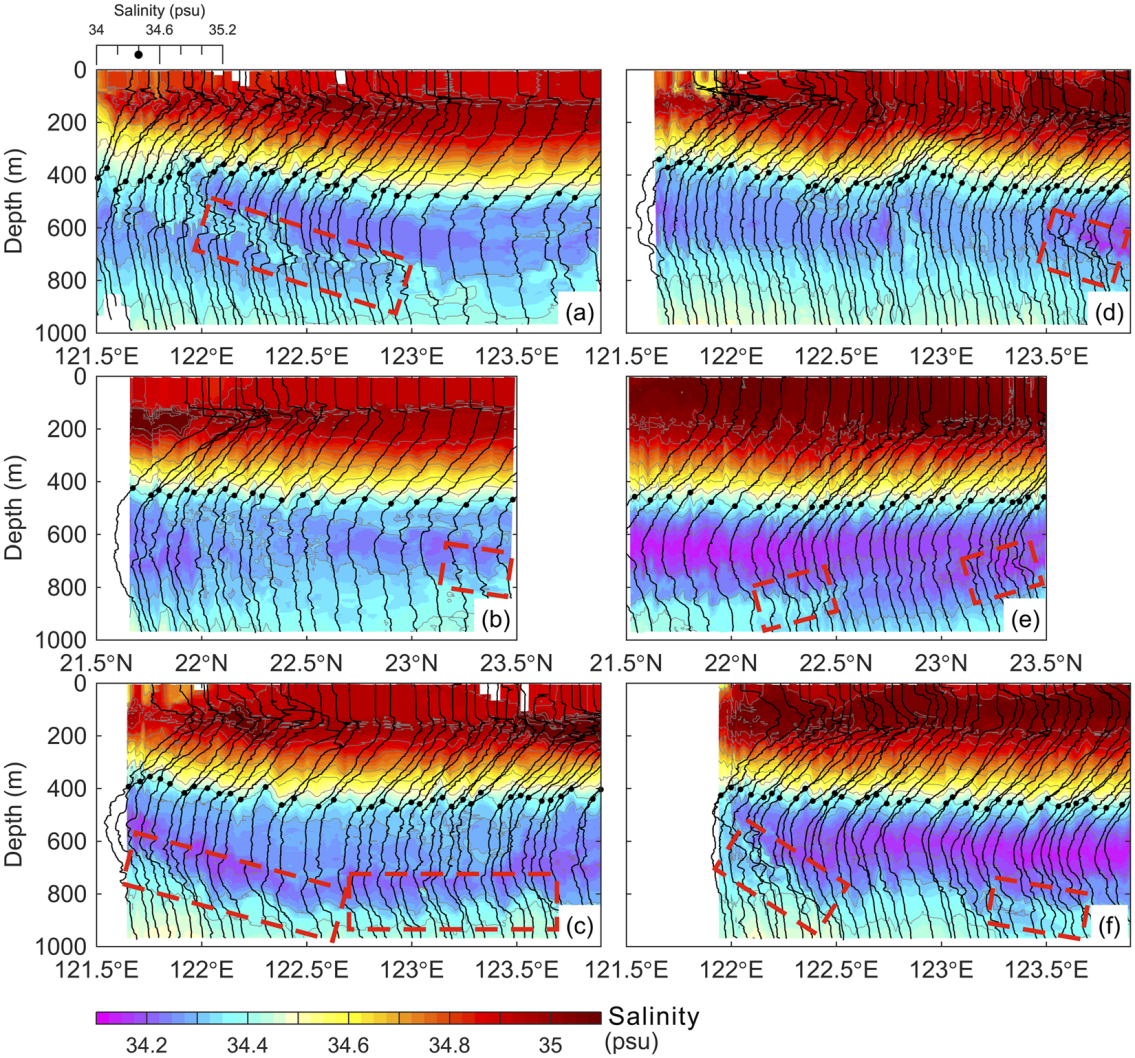
Salinity distribution (color shaded) in each transect of the glider observations. The surveys were during (**a**) 15 Dec. 2016–26 Dec. 2016 at N1, (**b**) 26 Dec. 2016–4 Jan. 2017 at N2, (**c**) 4 Jan. 2017–14 Jan. 2017 at N3, (**d**) 14 Jan. 2017–31 Jan. 2017 at N1, (**e**) 1 Feb. 2017–20 Feb. 2017 at N2, and (**f**) 20 Feb. 2017–5 Mar. 2017 at N3. The black curves are salinity profiles collected during each dive of glider samplings. A salinity profile scale is indicated in the upper left of (**a**). The black dots mark longitude at zonal section or latitude at meridional section and S = 34.40 of each salinity profile. The red dashed rectangles indicate locations of potential interleaving layers.

During the second survey, the water mass at N1 was mostly NPIW near the offshore end, tended to be KIW in the middle and was close to NPIW again near the onshore end of this section (Fig. 2d). It is plausible that the cyclonic eddy C2 in Fig. 1d played a certain physical role in affecting the spatial distribution of NPIW and KIW at N1. The salinity distribution in Fig. 3d (next subsection) shows that isohalines are elevated around 122.8°E because of the upwelling in the cyclonic eddy. Therefore, the intermediate water mass appeared to be KIW-like in the middle portion of N1 transect. It is clear that NPIW was the dominant water mass observed at the meridional transect N2. At N3, the dominant water mass was NPIW over the eastern half of the transect, and the water mass became the mixture of NPIW and KIW west of 122.5°E. The zigzag features remained in many T-S profiles, but the salinity anomaly value in a single zigzag was approximately 50% of that observed during the first survey. Since the surrounding outer ring of an anticyclonic eddy had reached the east and south sides of the triangle track during the second survey (Fig. 1e,f), the observation of very low ${S}_{{\rm{\min }}}$ there suggests that the eddy carried NPIW. This observation lends support to the viewpoint that an eddy carries water mass^25^.

### Interleaving layers in the salinity profiles

We only demonstrate glider observed salinity in the three transects in Fig. 3 because the interleaving feature is more distinct in the salinity than in the temperature profiles. Overall, the most noticeable interleaving appeared in 121.9–123°E and 123.3–123.9°E, within 500 to 800 m during the first survey at N1 (dashed red rectangle in Fig. 3a). The higher salinity layer (S > 34.32) intruded toward the east from ~122°E to 122.9°E (~100 km), and its thickness was ~180 m near 122°E and 20–50 m between 122.5 and 122.9°E, with smaller scale layered structures seen from the zigzag feature in salinity profiles. At the meridional transect N2, sporadic interleaving features with length scale smaller than those appeared at N1 are seen from Fig. 3b. At N3, no large scale interleaving layers such as those observed at N1 appeared in the salinity transect (Fig. 3c). Approximately one month later (Table 1), the glider reached the western corner of the triangle track again, and the repeated survey at N1 suggests that the salinity pattern in Fig. 3d differs from that in Fig. 3a, presumably due to the influence of the small cyclone (Fig. 1d). The isohalines within 200 and 450 m depths were heaved between 122.8° and 123.2°E. Comparing Fig. 3d with Fig. 3a, interleaving layers on the onshore half of N1 became blurred during the second survey. A very low salinity patch (S < 34.10) was observed at ~600 m near the easternmost part of N1, likely accompanied with shorter interleaving scale (Fig. 3d). The second survey at N2 and N3 observed that relatively low salinity waters occupied the intermediate layer from 550 to 750 m, which were presumably brought by the impinging anticyclone (Fig. 1e,f). Possible interleaving layers are seen in the intermediate layer between 22.2° and 22.5°N and between 23.3° and 23.5°N at N2 (Fig. 3e) and between 122° and 122.3°E and between 123.2° and 123.7°E at N3 (Fig. 3f). Overall, the salinity differences between these interleaving layers are from 0.05 to 0.10.

Note that the prevailing winds in our hydrographic survey region is the northeasterly monsoon in winter-like months from October to February and southwesterly monsoon in summer, with the former being stronger than the latter. During our glider observations, the mixing in the upper ocean was conceivably enhanced by the winter monsoon^26^ with a typical mean wind speed of 10 m s^−1^, which may produce an ~100 m thick mixed layer.

Despite the wind-induced influence on the salinity in the upper 100 m, the synoptic scale variation of salinity in the intermediate layer of the Kuroshio is primarily caused by the impingement of mesoscale eddies on the Kuroshio and, in turn, by their interactions^6,7,12^. The maximum salinity variation in the intermediate layer can reach 0.20, depending on the water mass carried by eddies from the east and the injection of SCSIW from the Luzon Strait south of Taiwan^6,7^. The duration of the change of salinity is the time scale of an eddy-Kuroshio interaction, which ranges from ~60 to 90 days^12^. Hence the upper bound for the uncertainty of salinity measurement in a zonal transect within 7 days is ~0.02, which is still less than the salinity differences between the observed interleaving layers 0.05–0.10.

## Discussion

**Figure 4:**
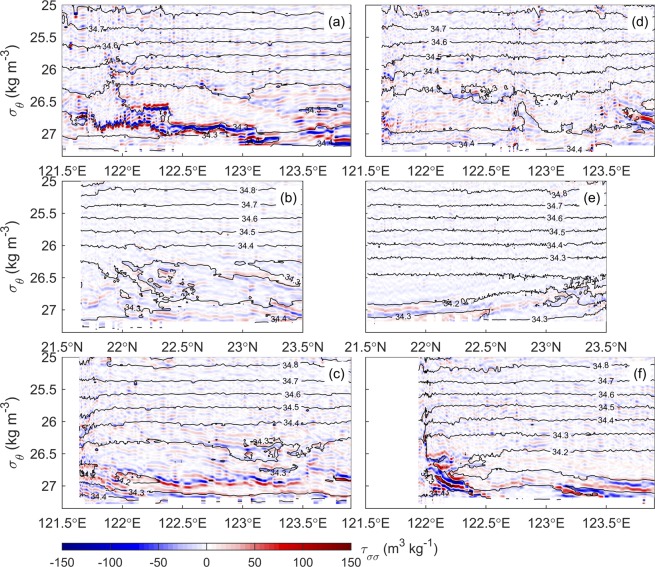
Diapycnal spiciness curvature ${{\rm{\tau }}}_{{\rm{\sigma }}{\rm{\sigma }}}$ calculated from the hydrographic data. The values of ${{\rm{\tau }}}_{{\rm{\sigma }}{\rm{\sigma }}}$ are illustrated in longitude (or latitude) versus potential density (25.0–27.4 kg m^−3^) plots at transects N1, N2, and N3 during the first survey (**a**–**c**) and the second survey (**d**–**f**). The black contours are isohalines.

To objectively represent the locations of interleaving layers, we adopted the diapycnal spiciness curvature (DSC, τ_σσ_) (Methods) as a measure of the lateral coherence and strength of interleaving layers. A high value of $|{{\rm{\tau }}}_{{\rm{\sigma }}{\rm{\sigma }}}|$ represents a clear diapycnal interface between two different water masses. As $|{{\rm{\tau }}}_{{\rm{\sigma }}{\rm{\sigma }}}|$ tends to be zero, it means that the diapycnal mixing diminishes the curvature of the vertical profile in the absence of horizontal advection^1^. Figure 4 shows ${{\rm{\tau }}}_{{\rm{\sigma }}{\rm{\sigma }}}$ in longitude (or latitude) versus potential density (25.0–27.4 kg m^−3^) plots of each transect. It is easily seen that the corresponding locations of relatively high absolute values of ${{\rm{\tau }}}_{{\rm{\sigma }}{\rm{\sigma }}}$ are primarily associated with the salinity transects in Fig. 3. The prominent interleaving mostly occurred along the σ_θ_ = 27.0 kg m^−3^ isopycnal during the observations of the first survey at N1 (Fig. 4a) and N3 (Fig. 4c) and the second repeated survey at N3 (Fig. 4f).

In addition to the stratification and the differences between diffusivities of momentum, temperature, and salinity, the growth of interleaving layers is conceivably affected by the background shear and density gradient in a highly dynamic environment such as the Kuroshio off the east coast of Taiwan. The influence of baroclinic and shear effects on the growth of interleaving intrusions has been examined with the consideration of baroclinic and sheared barotropic geostrophic fronts containing constant horizontal and vertical shears and a horizontal density gradient^27^. The velocity shear could tilt the slope of the interleaving layer and, in turn, influence the growth of the interleaving. The horizontal density gradient in the front could enhance or suppress the growth of the interleaving, depending on the isopycnal slope relative to the interleaving layer slope. The growth of the interleaving is enhanced when the interleaving layer is along the isopycnal slope and is suppressed when the interleaving slope opposes the isopycnal slope^27^, which are schematically illustrated in Supplementary Fig. S3.

The effect of the baroclinicity on the growth of interleaving layers in the Kuroshio is examined using the inverse growth rates of the fastest growing perturbation in a low-shear and high-shear environment, ${{\rm{\lambda }}}_{{\rm{l}}}^{-1}$ and ${{\rm{\lambda }}}_{{\rm{h}}}^{-1}$, respectively (Methods). To estimate the barotropic horizontal shear ${\bar{v}}_{x}$ in the intermediate layer of the Kuroshio, the velocities measured by a pair of lowered acoustic Doppler current profilers (LADCP; see Data) at the western six of the eight stations on the KTV1 line^6^ (Fig. 1), which is close to transect N1, were used, and the shear was calculated using the barotropic (depth-averaged) velocity difference and the distance between two adjacent stations. The mean barotropic shear is thus 7.39 × 10^−7^ s^−1^ with the local barotropic shear between two adjacent stations from −1.91 × 10^−6^ to 2.64 × 10^−6^ s^−1^ (Supplementary Fig. S4). Considering that diffusivities in the Kuroshio should be eddy-driven due to the dramatic influence of the frequent impinging eddies and the baroclinic shear, the turbulent Prandtl number is set to 1. With the horizontal and vertical properties of the observed interleaving layers in the intermediate layer at N1 in Table 2, the values of ${{\rm{\lambda }}}_{{\rm{l}}}^{-1}$ and ${{\rm{\lambda }}}_{{\rm{h}}}^{-1}$ are 2.1–3.7 and 42.0–145.5 d, respectively. Because the life time of the interleaving at each transect is hardly obtained by the glider observations, it is not easy to evaluate which of the two time scales is reasonable in this study. However, the two interleaving features observed during the two surveys at the same transect are dramatically different (e.g., Fig. 4a vs. 4d and 4c vs. 4f), and the times of the two surveys at the transect are ~30 days apart, which provides an upper bound on the resident time of an interleaving structure observed in a fixed transect. In addition to the time separation of the two surveys at a transect, the horizontal advection induced by the velocity in the intermediate layer (typically less than 0.1 m s^−1^) could shift interleaving features to the downstream for ~8.64 km per day, which suggests that the resident time of an interleaving structure in a transect may be even shorter. Hence, the time scale of 42.0–145.5 d for the growth of the interleaving layers at the N1 transect can be excluded, and it suggests that the high-shear limit is unlikely the condition in the intermediate layer of the Kuroshio. Moreover, the absolute value of mean barotropic shear ${\bar{v}}_{x}$ (~7.39 × 10^−7^ s^−1^) is much smaller than the fastest growth rate in the low-shear limit ${{\rm{\lambda }}}_{{\rm{l}}}$ (3.1–5.5 × 10^−6^ s^−1^). This quantity of ${{\rm{\lambda }}}_{{\rm{l}}}$ is obtained using its inversion (${{\rm{\lambda }}}_{{\rm{l}}}^{-1}$) defined in Methods and the values quantified from our glider observations in Table 2. This relationship suggests that the development of interleaving layers satisfies the low-shear condition. The density front of the Kuroshio potentially enhances the double diffusive process in the growth of interleaving layers. The same analysis as from the aforementioned procedures is applied to the observations at the N3 transect, and the result is consistent with those at the N1 transect.

**Table 2:** Horizontal and vertical properties quantified from the observations during the first survey at the intermediate layer of the N1 transect (500–800 m and 122.71°–122.51°E in Fig. 3a).

Parameter	Meaning	Mean value	Standard deviation
*f*	Inertial frequency at 23.5°N	5.82 × 10^−5^ s^−1^	—
${\bar{{\rm{S}}}}_{{\rm{x}}}$	Cross-front salinity gradient	−4.08 × 10^−7^ m^−1^	3.73 × 10^−7^
${\bar{{\rm{S}}}}_{{\rm{z}}}$	Vertical salinity gradient	1.87 × 10^−5^ m^−1^	2.30 × 10^−4^
ρ_0_	*In situ* density	1026.81 kg m^−3^	—
${\bar{{\rm{\rho }}}}_{{\rm{x}}}$	Cross-front density gradient	−4.00 × 10^−6^ kg m^−4^	0.72 × 10^−6^
${\bar{{\rm{\rho }}}}_{{\rm{z}}}$	Vertical density gradient	−2.10 × 10^−3^ kg m^−4^	0.39 × 10^−3^
${\gamma }_{f}$	Nondimensional salt fingering flux	0.75	—
ε_z_	Nondimensional quantity	1.8 × 10^−3^	—

The condition for the double-diffusive intrusions is further examined using the criterion^27^ $0\le \frac{k}{m}\le \frac{{{\rm{\varepsilon }}}_{{\rm{z}}}{\bar{{\rm{S}}}}_{{\rm{x}}}/{\bar{{\rm{S}}}}_{{\rm{z}}}+{\bar{{\rm{\rho }}}}_{{\rm{x}}}/{\bar{{\rm{\rho }}}}_{{\rm{z}}}}{1+{{\rm{\varepsilon }}}_{{\rm{z}}}}$ (where *k* is cross-front wavenumber, and *m* is vertical wavenumber). The criterion is applied to the hydrography at N1 obtained from the first survey. The upper bound of the criterion ranges in 1.2–2.9 × 10^−3^, which was calculated using the quantities in Table 2. To estimate *m*, the wavenumber spectrum of the salinity anomaly was calculated. The salinity anomaly ${\rm{S}}^{\prime} ({\rm{z}})$ was obtained by subtracting the background salinity $\bar{{\rm{S}}}({\rm{z}})$ from the observed salinity profile, i.e., ${\rm{S}}^{\prime} ({\rm{z}})={\rm{S}}({\rm{z}})-\bar{{\rm{S}}}({\rm{z}})$. The background salinity profile was calculated by applying a fourth-order Butterworth filter with a cutoff wavenumber of 0.0125 m^−1^ (wavelength of 80 m) to the vertical salinity data^18^. After these calculations, the spectral peak of the salinity wavenumber spectrum is located at approximately 0.0167 m^−1^ (i.e., vertical wavenumber *m*). The horizontal wavenumber *k* estimated from the interleaving features in Fig. 1a is ~1.0 × 10^−5^ m^−1^. Therefore, the maximum interleaving slope *k*/*m* is ~6 × 10^−4^, which is within the range of 0 and the upper bound of 1.2–2.9 × 10^−3^. This result indicates that the driving mechanism of the observed interleaving layers in the N1 section (Fig. 3a) can be attributed to the double-diffusive instability.

The impingements of cyclonic and anticyclonic eddies on the Kuroshio during the observations altered the density slope across the Kuroshio^8,12^. The variation in the density slope may provide extra horizontal density gradients that are either positive or negative to enhance or suppress the growth of interleaving layers in the cross-front direction as depicted in Supplementary Fig. S3. During the first survey, the western half of the anticyclonic eddy A1 (Fig. 1a) potentially enhanced the density slope across the Kuroshio, which was a positive effect on the growth of the observed interleaving layers at N1 and N3 (Supplementary Fig. S3a). The eddy condition was complicated during the second repeated survey. The western half of the small cyclone C2 on the transect N1 (Fig. 1d), which tended to weaken the density slope of the Kuroshio, could possibly inhibit the development of interleaving layers at N1 (Figs 3d and 4d). The following anticyclonic eddy A2 conceivably increased density slopes to its surrounding waters, which further influence the growth of the interleaving at the three transects. The inference merits further comprehensive field observations to verify.

**Figure 5:**
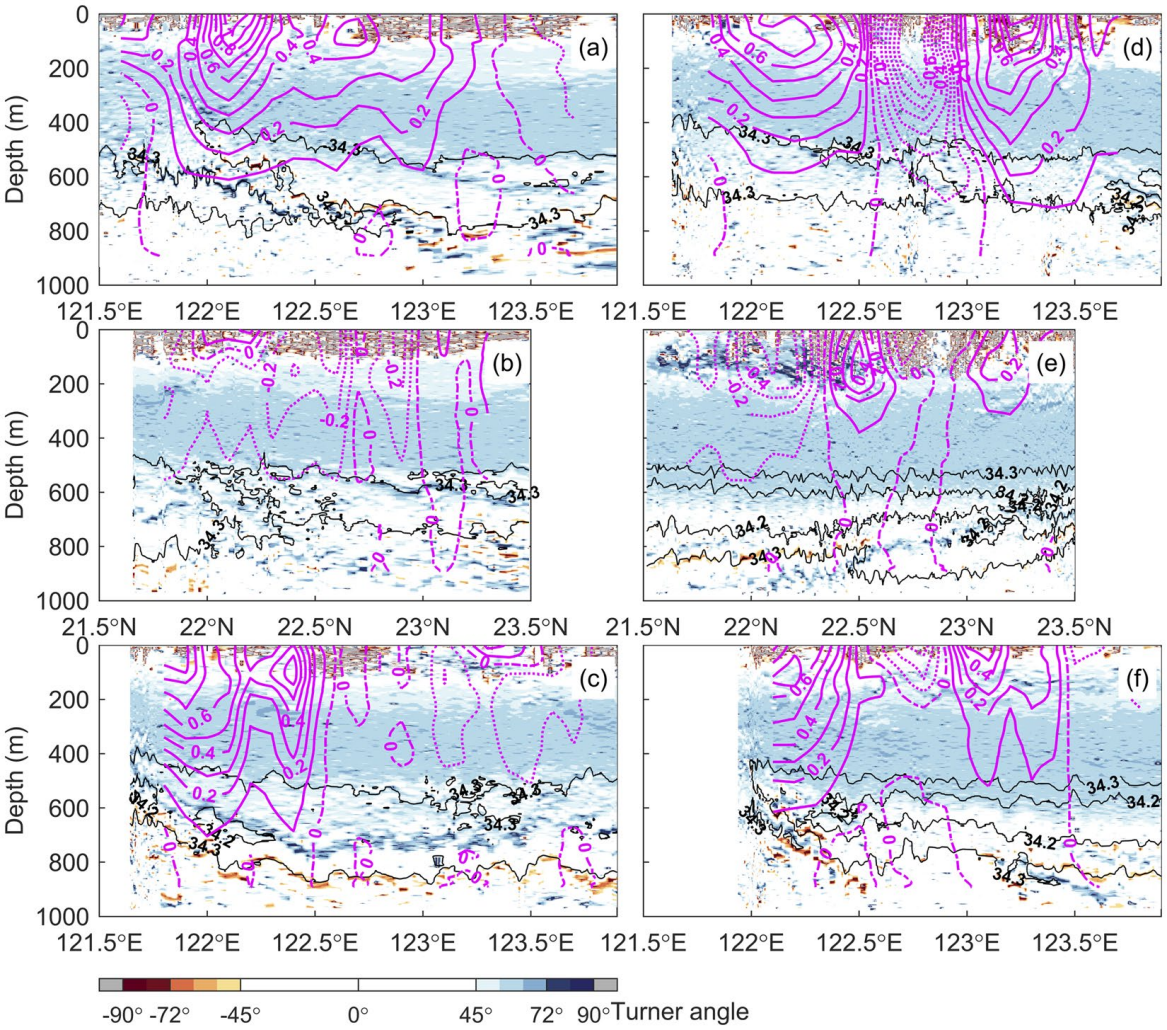
Turner angle (*T*_*u*_) and geostrophic velocity calculated from the observed hydrographic data. Results derived from the observations at N1, N2, and N3 are demonstrated in (**a**–**c**), respectively, for the first survey, and in (**d**–**f**), respectively, for the second survey. The black thin lines indicate isohalines of 34.2 and 34.3. The megenta solid and dashed lines are isotachs of geostrophic velocity in m s^−1^. The isotach interval is 0.1 m s^−1^.

The Turner angle *T*_*u*_^28^ (Methods) is helpful in analyzing stability and regimes of salt-fingering and diffusive convection in the observed interleaving layers. The values of *T*_*u*_ at each transect during each survey are illustrated in Fig. 5. The geostrophic velocities calculated from the hydrographic data at each transect through the thermal wind relationship are overlaid on Fig. 5. The stratification is in the (strong) diffusive convection regime as *T*_*u*_ is between −45° (−72°) and −90° and in the (strong) salt fingering regime between 45° (72°) and 90°. The stratification is stable as *T*_*u*_ is between −45° and 45° and statistically unstable as *T*_*u*_ is beyond ± 90°. Here, we focus on processes associated with the double-diffusive instability. The value of *T*_*u*_ indicates that most of the regions in each transect are subject to salt fingering. In the primary interleaving layers described based on Fig. 3, the salt fingering regime and diffusive convection regime alternately appeared in the interfaces. Accompanying the hydrographic structure of each transect in Fig. 3, the Turner angle further helps locate where double-diffusive processes are effective and dominating the development of the interleaving layers. As previously described, how the double-diffusive instability causes interleaving layers is schematically illustrated in Supplementary Fig. S1. The isotachs of geostrophic velocities also suggest that the primary interleaving layers in the intermediate layer were in weak flow region (<0.1 m s^−1^) of the Kuroshio.

Note that the double diffusive instability, including salt fingering and diffusive convection processes, is a necessary but not sufficient condition for the generation of interleaving layers. The strong current with velocity greater than 0.2 m s^−1^ in the upper 500 m (Fig. 5) and the weak salinity variation in the layer between 200 and 500 m (around T~15 °C and S~34.6 in Fig. 2) restrict the development of interleaving layers in a fixed transect.

Finally, whether the observed interleaving layers were caused by the advection of the cross-front near-inertial velocity perturbations or the inertial instability similar to the observations in the western Equatorial Pacific^18^ or in the Agulhas Current^22^ is discussed. Since there is no direct measurement of velocity with our glider observations, we are not able to examine the relationship between the salinity perturbations and the cross-Kuroshio velocity anomalies as done for the data set collected in the Agulhas Current^22^. However, in our observations, the maximum interleaving slope (*k*/*m*) satisfies the criterion of the double-diffusive instability as the primary cause of the interleaving, which suggests that the cross-Kuroshio velocity anomaly may be not effective. To verify this inference, the criterion for the effectiveness of inertial instability on the growth of interleaving layers^18^, i.e., *f*Q < 0, where Q (=$f+{\bar{v}}_{x}-{\bar{u}}_{y}-f{{\bar{v}}_{z}}^{2}/{{\rm{N}}}^{2}$) is the background potential vorticity, is further evaluated. The inertial frequency *f* is 5.82 × 10^−5^ s^−1^ (listed in Table 2) at our glider survey region. To our estimate in Discussion, the order of magnitude of the barotropic shear ${\bar{v}}_{x}$ is O(10^−7^–10^−6^) s^−1^, which is much larger than ${\bar{u}}_{y}$ and $f{{\bar{v}}_{z}}^{2}/{N}^{2}$ in the components of potential vorticity Q. Therefore Q must be positive and *f*Q > 0 in the Kuroshio. In this light, the cross-front velocity perturbations and the inertial instability are excluded from the primary driving mechanism of the interleaving in the Kuroshio.

In conclusion, the interleaving layer between dissimilar water masses has been observed in many low energy frontal zones in the world ocean. Our glider observations off the east coast of Taiwan also observed prominent interleaving layers beneath the Kuroshio primary velocity core (poleward velocity > 0.2 m s^−1^). The results from the high-resolution hydrographic data quantified the characteristics of the interleaving beneath the primary velocity core of the Kuroshio east of Taiwan. Not surprisingly, the main feature of these interleaving layers primarily appeared in the cross-front direction of the Kuroshio. In the intermediate layer in the Kuroshio region (approximately 500–800 m), KIW and NPIW intruded into each other, forming interleaving layers with horizontal scale of O(10–100) km and vertical scale of O(50) m. The resident time scale for the interleaving structures observed by the glider mostly at the N1 and N3 transects should be no longer than 30 days.

During the second survey at N2 and N3, the glider observed the western half of a westward propagating anticyclonic eddy that encountered the Kuroshio, and the characteristic of the water mass in the junction of the Kuroshio and the eddy more likely presented North Pacific Intermediate Water than that observed during the first survey on the eastern portion of the tracks. Although it is not the central focus of this study, the difference of water masses between the first and second surveys suggests that this anticyclonic eddy carried North Pacific water mass, which favors the concept of westward propagating mesoscale eddies that can trap and transport water masses^25^.

Since our glider survey was not a time-series observation, when and where the interleaving is initiated to perform is not resolved. It could be an evolving process during the observations or a result remaining after a previous event of eddy impingement on the Kuroshio. Moreover, the variations of the Kuroshio’s velocity structure, baroclinicity, and water mass composition are relatively energetic due to the frequent impingement of mesoscale eddies east of Taiwan and the input of South China Sea water through the Luzon Strait to the onshore side of the Kuroshio, which creates different conditions for the initiation and growth of the interleaving. Comprehensive, multiple platform observations using ships, gliders, and moored instruments are needed to better resolve the evolution of the interleaving of water masses in the Kuroshio.

## Methods

### Seaglider

The glider used in these observations is a Kongsberg Seaglider, which is equipped with conductivity, temperature, pressure, dissolved oxygen, fluorescence (chlorophyll), and backscatter optical sensors. Only the data collected by the first three sensors (i.e., CTD) were analyzed in this study. Seaglider is a buoyancy-driven autonomous underwater vehicle that is designed to glide from the ocean surface to 1000 m depth in a sawtooth pattern^29^. It steers through the water by controlling its pitch and roll and can navigate between waypoints to execute survey sections. Mission durations depend on payload, stratification, and profile depth but typically range from two to six months. Commanded remotely, gliders are able to acquire GPS position fixes and report their measurements via Iridium satellite telephone while at the sea surface (at the end of each dive cycle). Comparing distance travelled through the water with surface GPS position fixes allows estimation of depth-averaged current velocity. According to the report of Seaglider data quality control process (available at https://gliderfs2.coas.oregonstate.edu/sgliderweb/Seaglider_Quality_Control_Manual.html), the temperature measurement ranges from −2.5 to 43 °C and salinity ranges from 19 to 45. After quality control^30^, the accuracy is 0.001 °C for temperature and 0.01 (0.03 in strong thermocline regions) for salinity. The resolution of the sensor is 0.0001 °C for temperature and 0.001 for salinity. The salinity differences in the interleaving layers (Fig. 3) 0.05–0.10 are thus significant.

### Estimate of glider’s underwater location

A Seaglider also comprises built-in motion sensors, which provide pitch, roll, and heading angles while the glider is navigating in the water^29^. During the operation of the glider, a built-in microcomputer will calculate expected horizontal speeds and headings from its current position to the next target point. With the expected quantities of these parameters and the time interval between two adjacent CTD samplings, we can easily calculate the expected north-southward and east-westward displacements of the glider in one dive. When there is a background current, the glider can be drifted by the current and may therefore cause a drifting distance between the designate target point and the realistic position after one dive. The drifting speed, which is equivalent to a depth-averaged current, is obtained using the drifting distance and the time span of this dive. Furthermore, the drifting distance during two adjacent CTD samplings computed from the depth-averaged speed is added to the expected distance to obtain the coordinate of each CTD sampling. With this procedure, the integrated final coordinate should match the last surface position provided by the Global Positioning System (GPS). The coordinate of each salinity sample is used to plot the salinity transects in Fig. 1.

### Data

A Seaglider was launched off southernmost Taiwan on 8 December 2016 and was navigated to (121.51°E, 23.08°N) for hydrographic sampling along a triangular survey track covering the Kuroshio off the east coast of Taiwan (Fig. 1). The glider observations and associated study were sponsored by the Ministry of Science and Technology (MOST) of Taiwan under the Study of the Kuroshio – II (SK–II) project. After launching, the gilder was navigated to the western corner of the triangle track, and from there, the glider began to collect hydrographic data along the three sections (N1, N2, and N3) of the triangle successively (Fig. 1). Two repeated surveys for each transect were completed from 15 December 2016 to 5 March 2017, and 392 dives of hydrographic sampling (1 dive = 1 continuous dive and climb of the glider) were obtained during that period. The sampling period and number of dives at each transect are summarized in Table 3.

**Table 3:** Periods and sampling cycles of the Seaglider observations along the three sides of the triangular survey track.

Transect	Sampling period	Dives	Coordinate	
			Start point °E, °N	End point °E, °N
N1	15 Dec. 2016–26 Dec. 2016	56	121.51, 23.08	123.91, 23.49
N2	26 Dec. 2016–4 Jan. 2017	44	123.91, 23.44	123.95, 21.66
N3	4 Jan. 2017–14 Jan. 2017	51	123.90, 21.69	121.63, 23.14
N1	14 Jan. 2017–31 Jan. 2017	85	121.66, 23.16	123.91, 23.50
N2	1 Feb. 2017–20 Feb. 2017	93	123.91, 23.50	124.00, 21.51
N3	20 Feb. 2017–5 Mar. 2017	62	123.94, 21.52	121.94, 23.19

The LADCP used in the ship measurement at the KTV1 line (Fig. 1a) comprised two 300 kHz ADCPs with a downward-looking master and an upward-looking slaver, which was deployed with the CTD from the sea surface down to near the ocean bottom and then to the surface. The raw data were processed to be 8 m interval in the vertical for the whole water column (see Jan *et al*., 2015 for details). The processed data were depth- and time-averaged to obtain the time mean of barotropic velocity components in the zonal and meridional directions, and were further used to calculate horizontal shear of barotropic velocity (${\bar{v}}_{x}$) in the Kuroshio region east of Taiwan.

Near-real-time satellite altimeter sea surface height (SSH) and associated absolute geostrophic current data, which are produced by Ssalto/Duacs and distributed by Archiving, Validation, and Interpretation of Satellite Data in Oceanography (AVISO) at http://www.aviso.oceanobs.com/en/data/ products.html, were collected to supplement the interpretation of the glider data.

### Diapycnal spiciness curvature

In the water mass analysis, the spiciness is used as a state variable with the unit of kg m^−3^, which is a robust and sensitive indicator of how warm (spicy) and salty the water of a known density is, and a diagnosis of double diffusive instability and associated interleaving activity^1,31^. The spiciness is thus widely used to characterize water masses and their diffusive stability. The diapycnal spiciness curvature τ_σσ_ (DSC) is a second derivative of spiciness (τ) with respect to potential density along a profile^1^, which is calculated using

$$\frac{{{\rm{d}}}^{2}{\rm{\tau }}}{{{\rm{d}}{\rm{\sigma }}}^{2}}=\frac{{\rm{d}}[(1+{{\rm{R}}}_{{\rm{\rho }}})/(1-{{\rm{R}}}_{{\rm{\rho }}})}{{\rm{d}}{\rm{\sigma }}}$$

where τ is spiciness as defined by the relationship dτ = ρ(αdθ + βdS), σ is potential density, R_ρ_ is the vertical density ratio calculated from ${\rm{\alpha }}\frac{{\rm{d}}{\rm{\theta }}}{{\rm{dz}}}/{\rm{\beta }}\frac{{\rm{dS}}}{{\rm{dz}}}$, α is the thermal expansion coefficient (1.5 × 10^−4^ °C^−1^), β is the saline contraction coefficient (7.6 × 10^−4^ psu^−1^), and θ is potential temperature. The spiciness (τ) can be solved^31^, but in practice, τ is not necessarily solved to calculate ${{\rm{\tau }}}_{{\rm{\sigma }}{\rm{\sigma }}}$.

### Inverse growth rate of the fastest growing perturbation

The inverse growth rates of the fastest growing perturbation in low-shear and high-shear environments are, respectively, determined by^27^

$${{\rm{\lambda }}}_{{\rm{l}}}^{-1}=\frac{{\rm{2N}}}{{\rm{g}}}{(\frac{{{\rm{K}}}_{{\rm{M}}}}{{{\rm{K}}}_{{\rm{S}}}})}^{1/2}{|\frac{(1-{{\rm{\gamma }}}_{f}){{\rm{\beta }}\bar{{\rm{S}}}}_{{\rm{x}}}}{1+{(1+{{\rm{\varepsilon }}}_{{\rm{z}}})}^{1/2}}-\frac{{\bar{{\rm{\rho }}}}_{{\rm{x}}}}{{{\rm{\rho }}}_{0}}|}^{-1}$$

and

$${{\rm{\lambda }}}_{{\rm{h}}}^{-1}=\frac{8{{\rm{N}}}^{2}|f|}{{{\rm{g}}}^{2}}\frac{(1+{{\rm{\varepsilon }}}_{{\rm{z}}}){(1+{\bar{v}}_{x}/f)}^{1/2}}{{[(1-{\gamma }_{f}){{\rm{\beta }}\bar{{\rm{S}}}}_{{\rm{x}}}-{\bar{{\rm{\rho }}}}_{{\rm{x}}}/{{\rm{\rho }}}_{0}]}^{2}}$$

where ${\rm{N}}={(-{g\bar{{\rm{\rho }}}}_{{\rm{z}}}/{{\rm{\rho }}}_{0})}^{1/2}$, K_M_ is diffusivity of momentum, K_S_ is diffusivity of salinity, K_M_/K_S_ is the turbulent Prandtl number^32^, g (=9.8 m s^−2^) is the gravitational acceleration, ${\gamma }_{f}$ is a nondimensional ratio for salt fingering flux (normally 0.6–0.8), ρ_0_ is the *in situ* density, ${\bar{v}}_{x}$ is the horizontal shear of barotropic velocity, and ε_z_=(1 − γ_*f*_)/(R_ρ_ − 1) is a nondimensional quantity^33^. The quantity ε_z_ is a measure of the strength of the cross-front salinity gradient along isopycnals^18^.

### Turner angle

The Turner angle *T*_*u*_ is calculated by ${T}_{u}=ta{n}^{-1}(\frac{\alpha {{\rm{\theta }}}_{{\rm{z}}}+\beta {S}_{{\rm{z}}}}{\alpha {{\rm{\theta }}}_{{\rm{z}}}-\beta {S}_{{\rm{z}}}})$ ^28^.

## Supplementary information

Supplementary information for interleaving layers

## Data Availability

All data used for the figures and the analysis in this study have been provided either in Data or are available at the dataset of Mendeley through http://dx.doi.org/10.17632/ct5ppst6t2.2.
