# Supplementary material for: Glider observations of interleaving layers beneath the Kuroshio primary velocity core east of Taiwan and analyses of underlying dynamics
Source: Sci Rep. 2019 Aug 6;9:11401. doi: 10.1038/s41598-019-47912-z (PMC6684527; doi:10.1038/s41598-019-47912-z)
Supplement: Supplementary file 1 — Supplementary information for interleaving layers [file 41598_2019_47912_MOESM1_ESM.pdf]

Supplementary Information

Glider observations of interleaving layers beneath the Kuroshio primary velocity core east of Taiwan and analyses of underlying dynamics

Sen Jan*, Shih-Hong Wang, Kai-Chieh Yang, Yiing Jang Yang, Ming-Huei Chang
Institute of Oceanography, National Taiwan University
No. 1, Sec. 4, Roosevelt Rd., Taipei 10617, Taiwan
*Corresponding author, Email: senjan@ntu.edu.tw

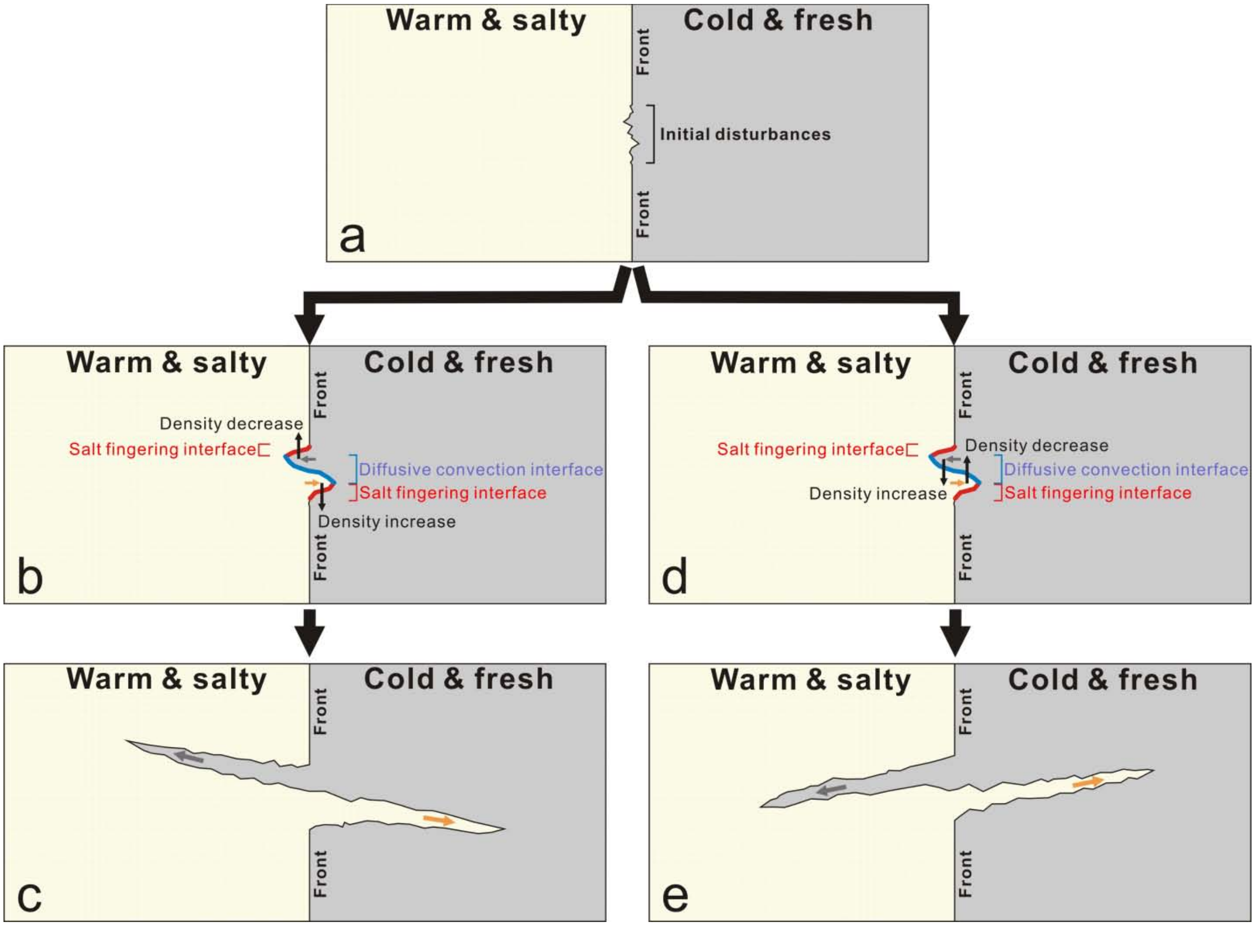

Supplementary Figure S1. Side-view of the development of interleaving layers caused by double-diffusive processes. As warm and saline (left) and cold and brackish (right) water masses encounter each other, initial disturbances in (a) can growth to be layered intrusions and therefore create diffusive convection interface (blue curve) and salt fingering interfaces (red curves) as shown in (b) or (d). If the double-diffusive instability in the front is dominated by diffusive convection (b), differences of the density flux at these interfaces lead to density decrease or increase in the intrusion waters, and, in turn, induce upward or downward motion as illustrated by black arrows in (b). These depth-varying double-diffusive buoyancy fluxes drive lateral interleaving motions further to each other in (c). If salt fingering is dominant in the variation of density flux (d), the resultant development of layered intrusions is shown in (e). The schematic plots are modified from Figure 1 of May and Kelley[28]. This figure is not covered by the CC BY licence. [© American Meteorological Society]. All rights reserved, used with permission.

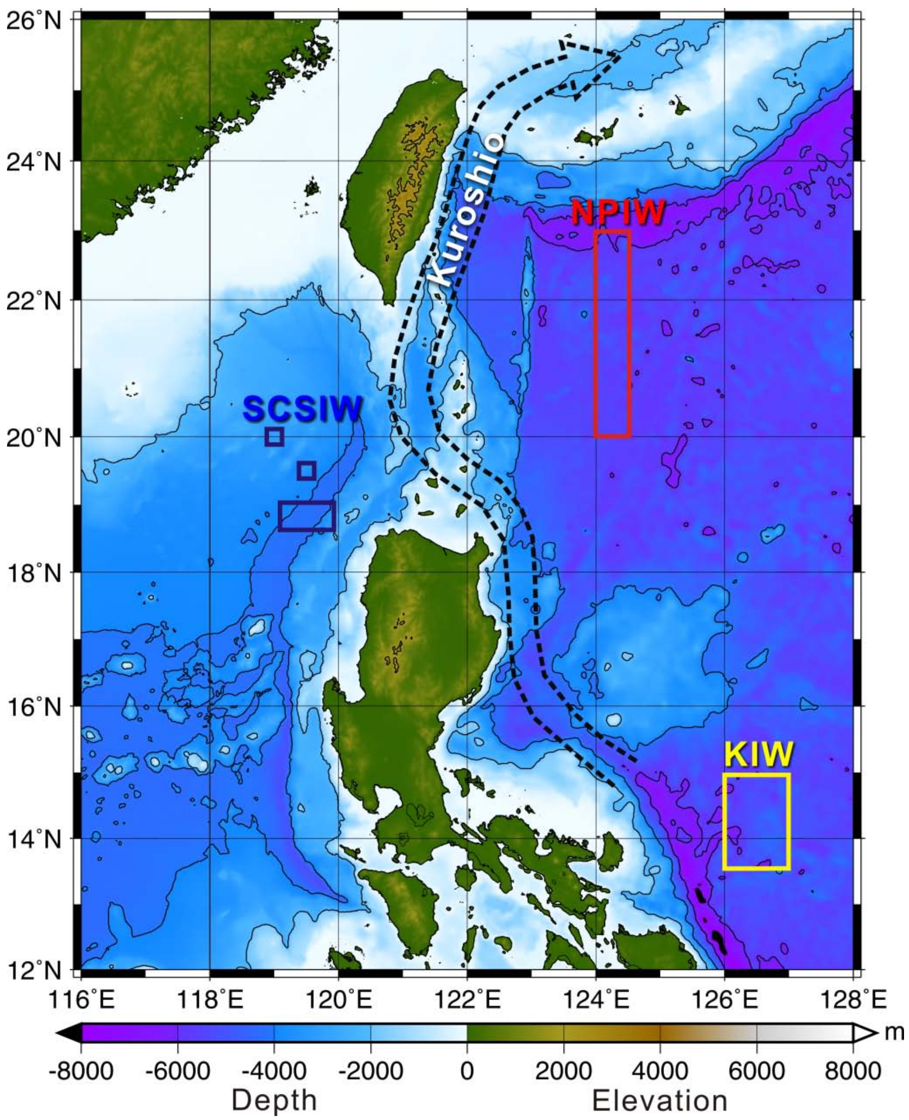

Supplementary Figure S2. Regions of selected historical CTD data[7] for calculating mean characteristic T-S curves. The yellow box indicates the region of CTD samplings for Kuroshio Intermediate Water (KIW), red box for North Pacific Intermediate Water (NPIW), and blue boxes for South China Sea Intermediate Water (SCSIW).

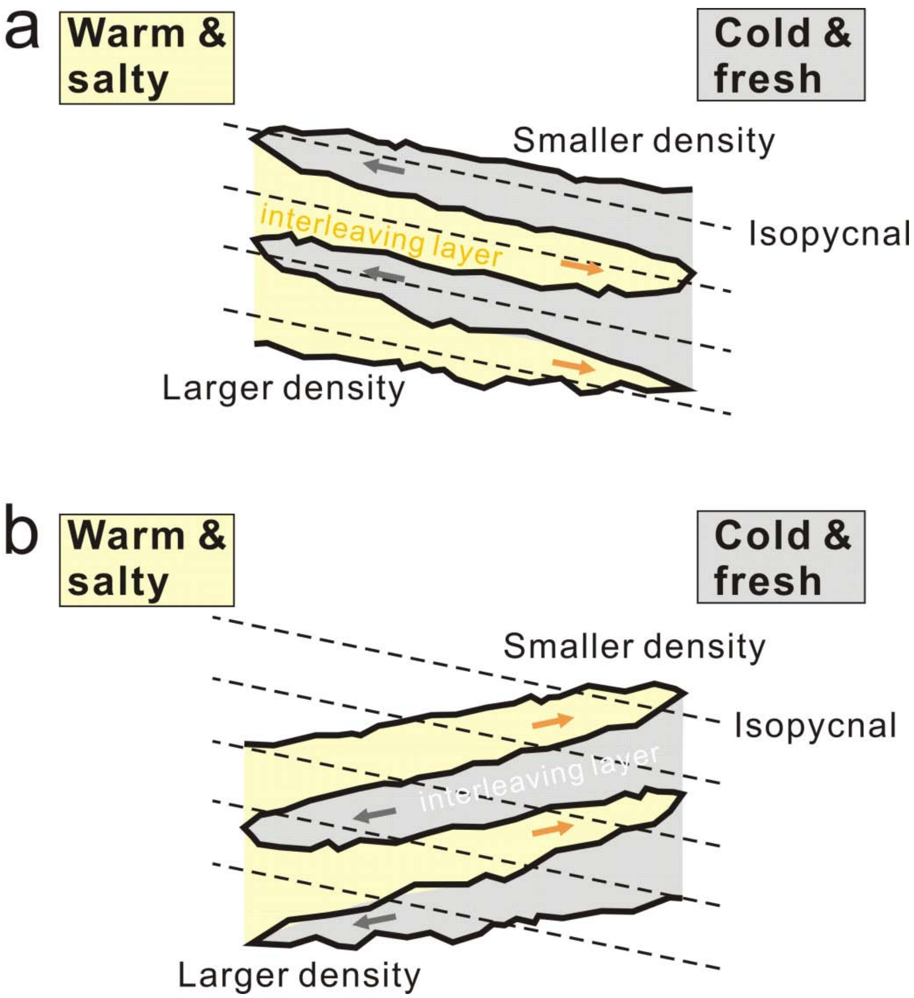

Supplementary Figure S3. Side-view of the influence of isopycnal slope on the growth of interleaving layers. Similar to the condition in Supplementary Fig. S1 but with a background baroclinic front, (a) if diffusive convection is dominant in the density flux across the interfaces and the isopycnal slope is in the direction of the interleaving layers, i.e., $\bar{\rho}_x < 0$ and $\bar{S}_x < 0$, the interleaving motion is enhanced; (b) if salt fingering is dominant in the density flux across the interfaces and the isopycnal slope increases density gradient along the interleaving layers, i.e., $\bar{\rho}_x < 0$ and $\bar{S}_x > 0$, the growth of interleaving needs to overcome the density gradient and the result is that the interleaving motion is suppressed[28]. This figure is not covered by the CC BY licence. [© American Meteorological Society]. All rights reserved, used with permission.

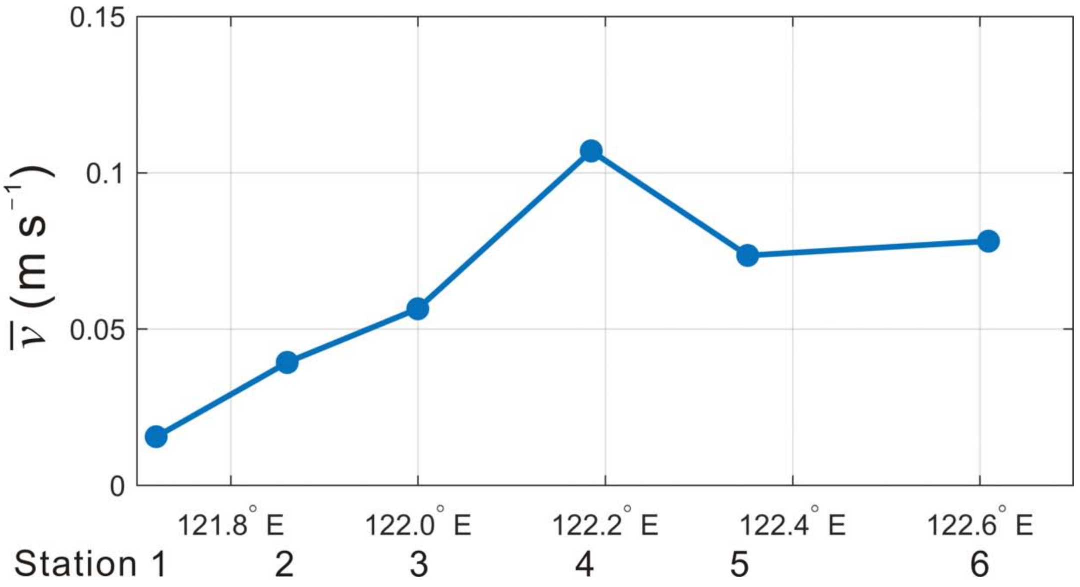

Supplementary Figure S4. Barotropic velocity at the western six of the eight hydrographic stations on the KTV1 line (Fig. 1a). The barotropic (depth-averaged) velocity was obtained by averaging the velocity profiles measured by Lowered acoustic Doppler current profiler during the nine ship surveys reported in Jan et al.[6]. The horizontal shear was therefore calculated using the barotropic velocity difference and the distance between adjacent stations, which ranges from -1.91×10^{-6} to 2.64×10^{-6} s^{-1}.
